# Factors Related to Mental Health of Foreign Care Workers in Long-Term Care Facilities in Japan during the COVID-19 Pandemic—A Comparative Study

**DOI:** 10.3390/ijerph192416491

**Published:** 2022-12-08

**Authors:** Qian Wu, Yuko Yamaguchi, Chieko Greiner

**Affiliations:** Graduate School of Health Sciences, Kobe University, 7-10-2 Tomogaoka, Suma, Kobe 6540142, Hyogo, Japan

**Keywords:** mental health, foreign care workers, workload, sense of coherence

## Abstract

This study aimed to evaluate the factors related to the mental health of foreign care workers in Japan’s long-term care (LTC) facilities and compare their results with those of native care workers. We conducted a cross-sectional survey covering 80 LTC facilities across Japan between August and November 2021. The survey mainly included mental health, workload, reward, sense of coherence, loneliness, COVID-19-specific factors and sociodemographic variables. The results show that workload was a distinct feature associated with the mental health of foreign care workers (n = 172) when compared with those of native care workers (n = 154). In addition, we found that the relationship between cultural adaptation and mental health in a sample of foreign care workers was mediated by loneliness and sense of coherence (SOC). Finally, we found that reward, loneliness, SOC, and COVID-19-specific factors had significant impacts on the mental health of both foreign and native care workers. These findings highlight the importance of support measures from the workplace for foreign care workers. Workplace interventions that focus on workload, reward, and sense of coherence strategies are required to address mental health improvement and may still be of value in dealing with the continuing COVID-19 pandemic.

## 1. Introduction

By 2050, the number of people in OECD countries who are aged 80 years or older will rise from about 5%, as it currently stands, to almost 10% on average [1]. However, the growth of long-term care (LTC) services has been slower than the growth of the elderly population. To address this issue, many countries regard attracting and employing immigrants as one viable option. According to statistical data in OECD countries, foreign care workers make up almost 20% of the population who worked in LTC facilities [2]. Japan is no exception. As a country with one of the fastest-aging populations in the world, about 8% of the population was aged 80 years or older in 2015, and that number is expected to increase substantially by 2050 [3]. To compensate for the labor deficit in long-term care institutions, the Japanese government has recruited foreign care workers under economic partnership agreements (EPA) with Asian countries, starting with Indonesia and followed by the Philippines (in 2009) and Vietnam (in 2014) [4]. In 2017, the policy-related care work visa was issued to stimulate an increase in foreign participation in the LTC industry [5]. According to government statistics, 43,446 foreign workers were employed in long-term care facilities in 2021, an increase of 18.0 percent from the previous year [6]. As the number of foreign care workers is growing annually, they have already assumed a significant role in caring for the elderly.

However, the complexity of LTC environments and intensive workloads have a negative impact on the mental health of foreign care workers, manifesting as anxiety and depression, for example [7]. Studies on foreign care workers reported that they have poor working conditions such as low wages, higher working hours, low job security and workplace abuse in host countries [8,9]. In addition, visa restrictions have been placed on foreign care workers. The majority of foreign care workers in Japan possess a visa that restricts their employment to the healthcare profession and limits their length of stay, hence increasing their stress levels [10,11]. In addition, governmental restrictions, such as the requirement for 2–5 years of experience working in Japan’s long-term care institutions, require them to exert greater effort than locals in order to get the national nursing qualification [4]. In a previous study, responses from 22.5% of foreign care workers suggested that they were at risk of developing mental health problems [12]. Mental health problems were associated with the high rate of turnover and work performance [13,14]. To improve qualities of care and maintain well-being in the workplace, interventions regarding care workers’ mental health should be implemented [15]. However, research thus far has primarily focused on the qualitative findings [7]; there has been little research on the mental health and related factors of foreign care workers serving the public.

The relevant factors of foreign care workers’ mental health are complex and diverse, and mainly close to their migration environment and work conditions [15]. In most previous studies, the relationship between mental health and coping skills has been investigated as well [16,17,18]. Therefore, the following hypotheses were established.

### 1.1. Work Conditions and Mental Health

There are several studies indicating the mental health of foreign workers associated with work conditions. Work-related stressors such as long working hours, high workload, and no free time were related to the mental health status among migrant workers in India [19]. Work overload stress of foreign care workers was the most common cause of mental health disorders [20]. According to a qualitative study, foreign care workers’ dissatisfactions regarding the workplace were due to the workload, constraints with time, and poor peer relations [21]. These factors might become work-related stressors of migrant workers, which result in mental stress [22]. Moreover, greater work-related stress results from perceived low rewards, which was identified as a risk factor of mental health among LTC workers [23]. In a previous study, Benjamin et al. found a correlation between the mental health of workers and the degree to which they had autonomy in their jobs, as well as interactions with coworkers and supervisors [24]. However, the specific factors of work conditions that are connected with mental health among foreign care workers have not been studied in depth in comparison to native care workers.

**Hypothesis** **1.** *Heavy workload is associated with negative mental health.*

**Hypothesis** **2.** *There is a relationship between reward and positive mental health.*

### 1.2. Sense of Coherence and Mental Health

There is empirical evidence of a strong relationship between mental health and sense of coherence (SOC), such that individuals with a strong SOC report a better overall health status [25]. It is a concept that reflects the ability to cope with stress and is at the core of the autogenesis theory [26]. In light of the current pandemic crisis, stress and challenges have been evident. In order to cope with them, SOC is a crucial aspect for mental health in care professionals who are exposed to stressful settings [27]. A solid SOC emerged as the strongest predictor for less severe symptoms of anxiety and depression among healthcare workers in Germany [28]. In Japan, it was identified that poor mental health was related to weak SOC among healthcare workers amid the COVID-19 pandemic [29]. It is extremely important that is an attempt is made to understand the connection between the mental health and SOC of foreign care workers because so little research has been carried out on this topic.

**Hypothesis** **3.** *Sense of coherence is associated with positive mental health.*

### 1.3. Loneliness and Mental Health

Immigrants’ social factors have been identified as important contributors to anxiety and depression symptoms [30]. Importantly, with a migrant background, most foreign care workers experienced a sense of loneliness [31]. Previous research also indicates that loneliness negatively affects the health and well-being of immigrants [32]. Additionally, the COVID-19 pandemic introduced global experiences of social isolation. Because of the great distance between Japan and their home countries, it is especially possible for foreign care workers to experience negative effects on their mental health while working in Japan.

**Hypothesis** **4.** *Loneliness was related to negative mental health.*

### 1.4. COVID-19-Specific Factors and Mental Health 

On March 2020, the World Health Organization (WHO) declared the rapid worldwide spread of coronavirus disease 2019 (COVID-19) to be a pandemic. Since the onset of the ongoing pandemic, numerous cluster outbreaks of COVID-19 have been reported in long-term care (LTC) facilities worldwide, affecting both the residents and the care staff [33]. In the Japan Geriatrics Society, LTC facilities are particularly vulnerable places with the majority of residents at high risk of complications. To date, it was estimated that 1,600 LTC facilities reported cluster infection cases due to the emergence of COVID-19 [34]. Caring for the elderly and for persons with physical and mental disabilities or pre-existing medical conditions, LTC workers are the first-line defense in protecting those most vulnerable to infections and disease. Their health and safety are directly related to the benefits of residents and facilities [35]. During the COVID-19 pandemic, the immigrant population in Norway reported high levels of depression and anxiety [36]. The COVID-19 outbreak could be considered an uncontrollable stressful life event that can contribute to the development of or increase in mental health problems in foreign care workers. There is little evidence about how COVID-19 has impacted foreign care workers in LTC facilities.

**Hypothesis** **5.** *COVID-19 specific factors are associated with mental health.*

### 1.5. Cultural Adaptation and Mental Health

Cultural adaptation is the process by which immigrants decide which parts of their culture from their home country to keep and which ones to change to fit their new surroundings [37,38,39]. These decisions might cause acculturative stress among immigrants. Studies in the U.S. have concluded that the mental health of immigrants was highly influenced by acculturative stress [40]. Research on foreign care workers also identified that cultural adaptation was an important predictor of mental health status [41,42]. Notably, according to the results of a survey regarding the problems foreign care workers face in Japan, they are experiencing difficulty with administrative procedures, real estate contracts, and automobile sales, as well as other procedures [43]. In addition, changes in temperature, the cost of living, culture, and language may make adaptation difficult for foreign workers who live abroad [44].

**Hypothesis** **6.** *Cultural adaptation is related to the mental health.*

At present, there is a worldwide increase in interest in foreign care workers’ difficulties and mental health. Based on the literature review, limited studies are available on comprehensive mental health support for foreign care workers. Additionally, little is known about the distinct characteristics of foreign care workers in long-term care facilities in comparison with native workers. To fill this gap, the goal of this study was to examine the factors that affect the mental health of foreign care workers in Japan’s LTC facilities and compare their results with those of native care workers.

## 2. Materials and Methods

### 2.1. Study Design

This quantitative study used a cross-sectional design. Data were collected using online questionnaires (Microsoft Forms). We followed the reporting guidelines of the Strengthening the Reporting of Observational Studies in Epidemiology (STROBE) statement for observational studies (Supplementary File S1).

### 2.2. Setting and Data Collection

Data collection took place between August and November 2021. The target population included foreign care workers and native care workers in long-term care facilities in Japan. We randomly selected 80 care facilities to distribute the study instructions as well as the questionnaire invitations. Care facilities which accepted EPA program were chosen from the latest open data on the Ministry of Health, Labour and Welfare website. The study was performed using an online survey addressed to care workers. The link to the questionnaire survey along with the introductory letter was sent to these facilities.

The participant selection criteria included: (1) working at the current facility for more than one month, (2) providing direct nursing care, except in cases where the participant was the facility head and (3) ability to read Japanese or English.

We developed separate questionnaires for foreign care workers and Japanese care workers, including demographic characteristics, work-related information (reward and workload), mental health and other variables. English and Japanese versions were available for foreign care workers and added extra items including cultural adaptation and migration-related characteristics.

### 2.3. Sample Size

The sample size was determined by using the G*Power software (v3.1.9.2, Heinrich-Heine-Universität Düsseldorf, Düsseldorf, Germany) [45,46]. We used 2-sided testing, odds ratio = 2, Pr (Y = 1|X = 1) H0 = 0.5, α err prob = 0.05, power (1-β err prob) = 0.85, R^2^ other X = 0.6. The minimum sample sizes for foreign and native care workers were 167 and 120, respectively.

### 2.4. Measurements

#### 2.4.1. Mental Health

Mental health was assessed using the Kessler 6 scale. This scale measures non-specific psychological distress and is utilized as a screening tool for serious mental illness in community-based samples [47]. The original version of the K6 was developed in English and then translated into Japanese [47]. The reliability and validity of the K6 have been systematically confirmed [47,48]. The Kessler 6 scale uses a 5-point Likert scale (ranging from 0 = none of the time to 4 = all of the time) to assess how often the respondent felt (a) nervous, (b) hopeless, (c) restless or fidgety, (d) so depressed that nothing could cheer them up, (e) that everything was a great effort, and (f) worthless over the past 30 days. Scale items were summed to achieve a score of up to 24, with higher scores on the Kessler 6 scale indicating a worse state of mental health [47,48].

#### 2.4.2. Workload

Workload was assessed using the short version of the effort–reward imbalance questionnaire. Workload was assessed by the effort sub-scale, which determines time pressure, interrupted work, and increased workload, with a total range from 3 to 12 [49,50]. Every item was scored on a 4-point Likert scale ranging from 1 (strongly disagree) to 4 (strongly agree). The validity and reliability of the Japanese version and English version were confirmed, with Cronbach’s α coefficient 0.74–0.89 [50,51].

#### 2.4.3. Reward

Reward was assessed using the short version of the effort–reward imbalance questionnaire, which includes a seven-item reward subscale [48,49]. Items were scored using a four-point Likert scale ranging from 1 (strongly disagree) to 4 (strongly agree), with higher scores indicating a positive perception of reward, including mutual respect and promotion. The validity and reliability of the Japanese version and English version were confirmed, with Cronbach’s α coefficient 0.79–0.89 [50,51]. Accordingly, this sample had a Cronbach’s α coefficient of 0.79. Subscale scores were determined by summing the seven items.

#### 2.4.4. Sense of Coherence (SOC)

Sense of coherence was measured by the Sense of Coherence (SOC-3) scale [52]. It measures SOC based on three items. It consists of three questions concerning manageability, meaningfulness and comprehensibility—factors representing one’s internal resources when coping with difficulties in life [52,53]. The questions were: “Do you usually see a solution to problems and difficulties that other people find hopeless?” (manageability), “Do you usually feel that your daily life is a source of personal satisfaction?” (meaningfulness), “Do you usually feel that the things that happen to you in your daily life are hard to understand?” (comprehensibility) [52,53]. The sum score of the SOC-3 ranged from 1 to 7, and higher values indicated a higher sense of coherence [53]. The Japanese version and English version of the SOC scale have been shown to have adequate reliability and validity [53,54]. In this study, the Cronbach’s α coefficient was 0.73.

#### 2.4.5. Loneliness

Loneliness was assessed using the UCLA Loneliness Scale (UCLA) [55]. The UCLA Loneliness Scale has shown satisfactory reliability (Cronbach’s alpha = 0.84) [56] and both concurrent and discriminant validity [57]. This scale contains 10 items that assess how frequently a participant has felt certain emotions, with values ranging from 1 = never, 2 = rarely, 3 = sometimes, to 4 = often [58]. The scale consists of 5 positively and 5 negatively scored items with a total score of 10–40 points. Higher scores indicate greater degrees of loneliness and there is no identified cut-off score that defines loneliness [55,58]. In Japan, the reliability and validity of the original version have been evaluated [58]. In this study, the Cronbach’s α coefficient was 0.88.

#### 2.4.6. COVID-19-Specific Factors

COVID-19-specific factors included income changes during the pandemic, COVID-19 clusters in the workplace, periodic PCR tests, whether they had been infected with COVID-19, and whether they had cared for elderly individuals with COVID-19.

#### 2.4.7. Cultural Adaptation

The cultural adaptation items were created by us, based on the Sociocultural Adaptation Scale [59]. Based on a survey on foreign care workers, from the 40-item scale, we chose 5 general-culture-relevant items [59]. These items were making friends, becoming used to Japanese food, disaster preparedness, house hunting, and valuing freedom. Participants were asked to indicate the extent to which each statement was true for them on a 5-point Likert scale (from 1 “No difficulty” to 5 “Extreme difficulty”).

### 2.5. Statistical Analysis

This study included both descriptive and inferential statistics. A Kolmogorov–Smirnov test was used to examine normality. The continuous variables with normal distribution were represented by the mean and standard deviation (SD), and the continuous variables with non-normal distribution were represented by the median. The distribution of categorical variables was expressed as a frequency and percentage. The distribution difference in the demographic characteristics, as well as other variables, between foreign care workers and native care workers were evaluated by a t-test, a Mann–Whitney U test, a chi-squared test or Fisher’s exact test. Correlations, alpha reliabilities (α) for each scale, and descriptive statistics were performed with R software.

Data were further analyzed using Partial Least Squares Structural Equation Modeling (PLS-SEM). SmartPLS v3.0 software was used, selecting 5000 samples for the bootstrapping procedure [60]. PLS-SEM analyses follow a two-step approach given by Hair et al. [61]. PLS-SEM is a non-parametric technique that takes advantage of the variance that can be explained in latent unobserved dimensions. Smart PLS-SEM requires less information about residual distributions, measurement scales, and sample sizes compared with the covariance-based SEM [62]. In PLS-SEM, relations between latent and manifest variables, as well as between latent variables (structural/inner model), are defined in the form of path models. The directional interpreted paths are represented by connecting arrows.

### 2.6. Ethical Considerations

This study was conducted upon receiving the approval of the Ethics Review Committee of the Kobe University department of Health Sciences, Japan (No.1014). After obtaining approval from all the ethics committees involved, we contacted the institutions which provided us with formal authorization. All the participants who met the inclusion criteria were invited to participate in the study. A complete description of this survey and informed consent forms were sent to participants prior to the questionnaires. After the participants selected “Yes,” data collection was able to continue. The participants responded to the questionnaires anonymously.

## 3. Results

In total, we recruited 335 care workers, including certified care workers and care assistants. Overall, 5 of those participating through web-based entries declined to participate. After data cleaning, we found that 4 entries were missing substantial data. We excluded these entries from further analysis, leaving 326 valid responses (foreign care workers = 172; native care workers = 154). Regarding the nationality of foreign care workers, most of them were Vietnamese (n = 54, 31.40%), followed by Indonesian (n = 50, 29.07%), and the remaining were of other nationalities (n = 68, 39.53%) (Supplementary File S2). Table 1 shows the comparisons of the demographic characteristics between the foreign care workers and native care workers. The total participants’ average age was 35.05 ± 9.35 years old. However, there was an age difference between foreign and native care workers. The mean age of foreign care workers was 29.06 ± 5.25 years old. Additionally, the mean age of native care workers was 41.73 ± 8.35 years old. Moreover, the number of years of care work experience in native care workers was 10.65 ± 5.72, which was much longer than foreign care workers. There were also significant differences in professional status between the two groups. Overall, 62.21% foreign care workers did not yet have the necessary national qualifications. On the contrary, 66.23% native care workers had obtained their national qualifications.

As shown in Table 2, no significant differences were observed between the foreign care workers and the native care workers regarding mental health. Foreign care workers had a higher level of SOC than native care workers. Regarding workload and reward, the workload of native care workers was greater than that of foreign care workers, as was the reward level. A drop in income due to COVID-19 was evident among native care workers. Other COVID-19-specific factors show no difference between the two groups.

The results of hypothesis testing among foreign care workers and native care workers are summarized in Table 3. After controlling for demographic factors (age and professional status), we found that the impact of COVID-19 was the strongest factor that positively affected mental health among both foreign care workers (β = 0.325, *p* < 0.001) and native care workers (β = 0.316, *p* < 0.001). It was also shown that loneliness has a significant effect on the mental health of both foreign care workers (β = 0.246, *p* < 0.001) and native care workers (β = 0.220, *p* = 0.001). Meanwhile, mental health was significantly associated with reward (FCW: β = −0.112; NCW: β = −0.195) and SOC (FCW: β = −0.232; NCW: β = −0.293). Workload only had a significant impact on the mental health of foreign care workers (β = 0.198, *p* < 0.001).

The PLS analysis for foreign care workers is shown in Table 4. The relationship between workload and mental health (β = 0.247, *p* < 0.001) is statistically significant, in line with the results shown in Table 3. Additionally, reward was found to have a negative effect on mental health (β = −0.114, *p* = 0.023). Conversely, cultural adaptation had no direct effect on the mental health of foreign care workers (β = 0.038, *p* = 0.460). Finally, by looking at the internal variance inflation factor (VIF) index to determine the multicollinearity between latent variables, we can see that all values were lower than the standard value of 5.00 (inner VIF: 1.200–1.640). Therefore, multicollinearity was not confirmed [63]. As a result, Hypothesis 6 was rejected, and Hypotheses 1, 2, 3, 4, and 5 were accepted.

In this study, there were two indirect effects, which are given in Table 5. The first indirect effect concerns the mediating role of loneliness in the relationship between mental health and cultural adaptation. This hypothesis is supported, according to the following results: t = 2.890, *p* = 0.004. The second indirect effect concerns the mediating role of SOC in the relationship between mental health and cultural adaptation. This hypothesis is supported, according to the following results: t = 2.545 and *p* = 0.011.

## 4. Discussion

The present study investigated mental health and related factors in foreign care workers and compared these factors with those of native care workers in Japan. To the best of our knowledge, this is the first study to compare the mental health outcomes of foreign care workers and native care workers in long-term care (LTC) facilities. This study produced three key findings. First, compared with native care workers, we determined that workload was a distinct feature connected with the mental health of foreign care workers. Second, we found that the relationship between cultural adaptation and mental health in a sample of foreign care workers is mediated by loneliness and SOC. Thirdly, COVID-19-specific factors had a strong impact on the mental health of both foreign and native care workers. In addition, we have found that reward, loneliness, and SOC are significant factors associated with the mental health of both foreign and native care workers. In terms of cultural adaptation, there is no direct evidence linking the mental health of foreign care workers.

Regarding workload, we found that foreign care workers were prone to having a lower external workload than native care workers in LTC facilities in Japan. This finding may be explained by the difference in professional status between the two groups. A majority of foreign care workers are care assistants without a national qualification related to care work, whereas most native care workers are certified care workers. In Japan, most certified care workers have more than 3 years of care work experience and can undertake more specific age-related care, such as dementia care [64]. Care assistants, on the other hand, can only carry out daily life care for older individuals; for instance, they can help with bathing [65]. The age difference between local and foreign care workers may also play a role in the noticeable variation in workload. Consistent with prior research [66,67], there was a substantial age disparity between foreign care workers and local care workers in our study. Regarding physical workload, such as transferring a patient, younger care workers may feel less burdened [68]. In addition, owing to the status of foreign care workers, some leadership responsibilities, such as training new care employees, may not be assigned to them [7].

In the hypothesis testing, our findings demonstrated that only foreign care workers with a heavier workload are more likely to experience mental health issues. This was consistent with a study on the mental health of immigrant workers, which indicated that bad working conditions, particularly heavy workloads, were strongly related to mental illness [23]. Contrary to the findings of the care staff study [69], we did not find a relationship between the workload and mental health of native care workers. There is a rational explanation for this, in that immigrant workers’ mental health declines as a result of the stress brought about by growing workloads and low levels of reward in host countries [70]. Work-related stress has long been recognized as a key risk factor for migrant workers’ mental health [71]. An interesting finding from a study of interviews with foreign care workers in Japan is that completing paper care records and other documents in Japanese is also seen as a heavy workload that can lead to burnout [7]. However, the focus of this study was not the types of workloads in the LTC sectors, but rather on the relationships between mental health and workload.

Another finding is that both foreign and native care workers with a positive perception of reward tend to possess better mental health. This finding ties well with previous studies [21,72]. This is because increasing the appropriateness of rewards can make people feel positive emotions that are good for their mental health and well-being [24]. To be more specific, occupational rewards can be broken down into three categories: satisfaction with earnings, esteem, and job security [50]. There is a tendency toward declining mental well-being after migration among foreign care workers living with lower economic conditions [10]. This is because most foreign workers emigrated to developed countries for a better economic life [12,73]. In comparison with the high cost of living in Japan, foreign care workers are typically dissatisfied with their low pay, which may have a negative impact on their mental health [7]. A new study has also identified a correlation between poor self-esteem at work and mental health issues [74]. Many care workers have an esteem need for clear acknowledgement of their sacrifices and extraordinary efforts [75]. Meanwhile, lack of job security has been highlighted as a significant mental health risk factor for care workers during the COVID-19 pandemic [76]. Fear of becoming infected may make care workers feel unsafe at work, which could worsen their mental stress [77].

Although we assumed that cultural adaptation has a direct effect on mental health based on previous reports showing that such cultural-related stress facilitates poor mental health among migrant care workers [41,42], our results did not support this assumption. On the other hand, we found that cultural adaptation could have an indirect effect on mental health through the mediating effects of loneliness and sense of coherence (SOC).

Regarding loneliness, it has been defined as an unpleasant experience caused by a person’s assessment that their network of social relations is insufficient [78]. Our findings supported the psychological pathway of the conceptual model proposed by Berkman et al. [42], who argued that social relationships may influence mental health outcomes via multiple mechanisms. As one of the most significant post-migration stressors [30], loneliness was strongly associated with cultural adaptation among foreign care workers, and was further related to their mental health.

It was also found that the SOC completely mediated the effect of cultural adaptation and mental health. This was consistent with previous studies on immigrants [79]. Our result also identified a strong relationship between SOC and mental health status which is directly in line with previous findings [28,29]. By identifying the fact that SOC mediates the relation between cultural adaptation and mental health, more emphasis can be placed on utilizing interventions that strengthen SOC for the migration population with growing mental health disorders [26]. This may be particularly beneficial in terms of mental health policy, since it may suggest that governments and institutions offer multiple means of supports rather than relying solely on language and training in caregiving skills [80].

COVID-19-specific factors were found to be strongly associated with both foreign and native care workers’ mental health, even after controlling for demographic features. Research on the mental health status of care workers has largely focused on the associations between workplace safety [81] and work-related changes by COVID-19 [82]. Many scholars found that the worst mental health among care workers was associated with caring for COVID-19-infected patients [83,84] and the fear of infection [85]. A Japanese longitudinal study also revealed that the effects of COVID-19 may have substantially reduced their income, thereby threatening their mental health [86]. Importantly, many long-term care facilities have experienced COVID-19 outbreaks [87,88], and were associated with some significant drop-in care workers, owing to elevated absences and departures [89]. Therefore, the mental health of care workers may be negatively impacted by the stress caused by insufficient manpower and increased workloads [90].

This study possesses several limitations. First, due to the nature of cross-sectional study data collection, causal relationships between factors and mental health cannot be inferred. Second, the data collected were only from the early phase of the pandemic, which may not represent other periods of the pandemic in which circumstances might have changed. Thirdly, this study tested the mediating effects of loneliness and SOC on the relationship between cultural adaptation and mental health. However, several other factors, such as gender and social support, may act as moderators that may affect the tested relationship. Our survey was only available in Japanese and English. This limitation affected our participants because it was difficult for foreign care workers with less than N2 Japanese proficiency to participate in the Japanese version. In addition, there are a large number of Chinese and Vietnamese care workers in Japan. Since English is not their first language, they are ineligible to participate in our studies if they cannot comprehend English well. Therefore, it is strongly suggested that the scope of this study be broadened in the future by including multiple languages among the questionnaires.

## 5. Conclusions

These findings highlight the importance of support measures from the workplace for foreign care workers. Workplace interventions that focus on workload, reward, and sense of coherence strategies are required in order to improve workers’ mental health, and may still be of value in dealing with the continuing COVID-19 pandemic.

## Figures and Tables

**Table 1:** Demographic characteristics of care workers (n = 326).

	All	Foreign Care Workers	Native Care Workers	
Characteristics	n = 326	n = 172	n = 154	*p*-Value
	%/M ± SD	%/M ± SD	%/M ± SD	
Age	35.05 ± 9.35	29.06 ± 5.25	41.73 ± 8.35	<0.001 ^a^
Year of Care work experience	6.92 ± 5.61	3.58 ± 2.61	10.65 ± 5.72	<0.001 ^a^
Gender				0.648 ^b^
Female Male	220 (67.48) 106 (32.51)	118 (68.60) 54 (31.40)	102 (66.23) 52 (33.77)	
Marriage				<0.001 ^b^
Unmarried Married	181 (55.52) 145 (44.48)	115 (66.86) 57 (33.14)	66 (42.86) 88 (57.14)	
Professional status				<0.001 ^b^
Certified care worker Care assistant	167 (51.23) 159 (48.77)	65 (37.79) 107 (62.21)	102 (66.23) 52 (33.77)	
Workplace				0.498 ^b^
Special elderly nursing home Long-term care health facility	165 (50.61) 161 (49.39)	84 (48.84) 88 (51.16)	81 (52.60) 73 (47.40)	
Educational background				<0.001 ^c^
High school or technical school Junior college Bachelor degree Master degree	36 (11.04) 173 (47.79) 114 (34.97) 3 (0.92)	2 (1.16) 94 (54.65) 73 (42.44) 3 (1.74)	34 (22.07) 79 (51.30) 41 (26.62) 0 (00.00)	
Night shift				0.487 ^b^
Yes No	216 (66.26) 110 (33.74)	111 (64.53) 61 (35.47)	105 (68.18) 49 (31.82)	

Notes: M = mean, SD = standard deviation; ^a^: Mann–Whitney U test; ^b^: Chi-square test; ^c^: Fisher’s exact test.

**Table 2:** Average scores of variables in foreign care workers and native care workers.

	Foreign Care Workers	Native Care Workers	
Variable	n = 172	n = 154	*p*-Value
	M ± SD	M ± SD	
Mental health	8.18 ± 3.87	8.70 ± 4.22	0.245 ^d^
SOC	15.03 ± 2.82	13.91± 3.37	0.001 ^a^
Workload	8.35 ± 1.93	8.90 ± 2.16	0.014 ^a^
Reward	19.20 ± 3.88	20.76 ± 3.67	<0.001 ^d^
Loneliness	22.45 ± 4.89	22.95 ± 4.86	0.346 ^a^
COVID-19-specific factors Periodic PCR test in workplace			0.464 ^b^
Yes No	148 (86.05) 24 (13.95)	128 (83.12) 26 (16.88)	
COVID-19 outbreak in workplace			0.793 ^b^
Yes No	35 (20.35) 137 (79.65)	37 (24.02) 117 (75.98)	
Income changes during COVID-19			<0.001 ^c^
Increased a lot Increased Increased a little No change Decreased a little Decreased Decreased a lot	1 (0.58) 4 (2.32) 36 (20.93) 113 (65.70) 11 (6.40) 4 (2.33) 3 (1.74)	1 (0.65) 7 (4.55) 15 (9.74) 92 (59.74) 33 (21.43) 6 (3.90) 0 (0.00)	
Infected with COVID-19			0.225 ^c^
Yes No Unsure	9 (5.23) 155 (90.12) 8 (4.65)	4 (2.60) 142 (92.21) 8 (5.19)	
Cared for elderly with COVID-19			0.057 ^b^
Yes No	25 (14.53) 147 (85.47)	35 (22.73) 119 (77.27)	

Notes: M = mean, SD = standard deviation; SOC = sense of coherence; ^a^: Mann–Whitney U test; ^b^: Chi-square test; ^c^: Fisher’s exact test; ^d^: Student’s t- test.

**Table 3:** Factors associated with mental health among foreign care workers and native care workers.

	Foreign Care Workers			Native Care Workers		
Variable	n = 172			n = 154		
	Beta(β)	T-Value	*p* Values	Beta(β)	T-Value	*p* Values
SOC	−0.232	3.613	<0.001	−0.293	4.009	<0.001
Workload	0.198	3.739	<0.001	0.050	0.751	0.453
Reward	−0.112	2.120	0.035	−0.195	2.322	0.021
Loneliness	0.246	3.846	<0.001	0.220	3.297	0.001
COVID-19	0.325	6.263	<0.001	0.316	5.669	<0.001
	Adjusted R^2^ (Mental health) = 0.617			Adjusted R^2^ (Mental health) = 0.575		

Notes: SOC = sense of coherence; COVID-19 = COVID-19-specific factors.

**Table 4:** Hypothesis testing of mental health model in foreign care workers (n = 172).

	Hypotheses	Beta(β)	T-Value	95%CI	f^2^	*p* Values	VIF
H1	Workload → Mental health	0.247	4.683	[0.147,0.347]	0.132	<0.001	1.170
H2	Reward → Mental health	−0.114	2.139	[−0.228,0.001]	0.023	0.030	1.410
H3	SOC → Mental health	−0.239	3.828	[−0.366, −0.111]	0.097	<0.001	1.490
H4	Loneliness → Mental health	0.207	3.194	[0.081,0.334]	0.066	<0.001	1.640
H5	COVID-19 → Mental health	0.327	6.822	[0.235,0.419]	0.211	<0.001	1.280
H6	CA → Mental health	0.038	0.783	[−0.057,0.134]	0.003	0.460	1.200

Notes: SOC = sense of coherence; COVID-19 = COVID-19-specific factors; CA: Cultural Adaptation.

**Table 5:** Direct and indirect effects predicting mental health of foreign care workers (n = 172).

Path	Beta	T-Value	95%CI	*p* Values
Workload → Mental health	0.241	4.788	[0.131, 0.330]	<0.001
Reward → Mental health	−0.117	2.187	[−0.245, −0.035]	0.029
SOC → Mental health	−0.246	3.987	[−0.368, −0.119]	<0.001
Loneliness → Mental health	0.213	3.491	[0.076, 0.323]	0.001
COVID-19 → Mental health	0.329	6.538	[0.228, 0.424]	<0.001
Cultural Adaptation → Loneliness	0.362	6.094	[0.269, 0.493]	<0.001
Cultural Adaptation → SOC	−0.286	4.332	[−0.436, −0.178]	<0.001
Cultural Adaptation → Loneliness → Mental Health	0.077	2.890	[0.024, 0.125]	0.004
Cultural Adaptation → SOC → Mental Health	0.071	2.545	[0.023,0.127]	0.011
	Adjusted R^2^ (Mental health) = 0.591			

Notes: SOC = sense of coherence; COVID-19 = COVID-19-specific factors.

## Data Availability

Not applicable.

## Supplementary Materials

The following supporting information can be downloaded at: https://www.mdpi.com/article/10.3390/ijerph192416491/s1, Supplementary File S1 and Supplementary File S2.
