# Supplementary material for: Factors Related to Mental Health of Foreign Care Workers in Long-Term Care Facilities in Japan during the COVID-19 Pandemic—A Comparative Study
Source: Int J Environ Res Public Health. 2022 Dec 8;19(24):16491. doi: 10.3390/ijerph192416491 (PMC9779326; doi:10.3390/ijerph192416491)
Supplement: Supplementary file 1 [file ijerph-19-16491-s001.zip › Supplementary File S2.pdf]

Supplementary File S2

Table S1. Characteristics of foreign care workers (*N*=172) .

**Items**	**Foreign care workers n (%)**
**Nationality**	
Vietnamese	54 (31.40)
Indonesian	50 (29.07)
Philippine	38 (22.09)
Nepalese	14 (8.14)
Chinese	12 (6.98)
Brazilian	4 (2.33)
**Japanese Language Proficiency Test†**	
N1	23 (12.79)
N2	61 (35.47)
N3	75 (43.60)
N4	11 (6.40)
Unknown	2 (1.16)
**Satisfaction with migration**	
Extremely satisfied	20 (11.63)
Somewhat satisfied	96 (55.81)
Neither satisfied nor dissatisfied	46 (26.74)
Somewhat dissatisfied	8 (4.65)
**Feeling discriminated as a foreigner**	
Never	47 (27.33)
Seldom	99 (57.56)
Sometimes	26 (15.12)

† The Japanese-Language Proficiency Test is conducted both in Japan and outside Japan to evaluate and certify the language proficiency of primarily non-native speakers of Japanese since 1984. N4 and N5 measure the level of understanding of basic Japanese mainly learned in class. N1and N2 measure the level of understanding of Japanese used in a broad range of scenes in actual everyday life. N3 is a bridging level between N1/N2 and N4/N5.

Table 7 Cultural adaptation of foreign care workers. (*N*=172)

Items	Foreign care workers n (%)
**Getting used to the food in Japan**	
Extremely difficult	14 (8.14)
Difficult	14 (8.14)
Moderately difficult	40 (23.26)
A slightly difficult	88 (51.16)
Easy	16 (9.30)
**Making friends in Japan**	
Extremely difficult	42 (24.42)
Difficult	37 (21.51)
Moderately difficult	57 (33.14)
A slightly difficult	23 (13.37)
Easy	13 (7.56)
**Disaster preparedness in Japan**	
Extremely difficult	7 (8.14)
Difficult	27 (8.14)
Moderately difficult	40(23.26)
A slightly difficult	93(51.16)
Easy	5 (9.30)
**House-hunt in Japan**	
	9 (5.23)
Extremely difficult	25 (14.53)
Difficult	36 (20.93)
Moderately difficult	87 (50.58)
A slightly difficult	15 (8.72)
Easy	
**Worshipping in your usual way in Japan**	
Extremely difficult	5 (2.91)
Difficult	14 (8.14)
Moderately difficult	19 (11.05)
A slightly difficult	80 (46.51)
Easy	54 (31.39)
